# Postnatal care service utilization and associated factors among women who gave birth in Debretabour town, North West Ethiopia: a community- based cross-sectional study

**DOI:** 10.1186/s12884-018-2138-x

**Published:** 2018-12-27

**Authors:** Kihinetu Gelaye Wudineh, Azezu Asres Nigusie, Shumiye Shiferaw Gesese, Azimeraw Arega Tesu, Fentahun Yenealem Beyene

**Affiliations:** Department of Midwifery, College of Medicine and Health Sciences, Bahir Dar University, Bahir Dar, Ethiopia

**Keywords:** Utilization, Postnatal care, Maternal death

## Abstract

**Background:**

World health organization stated that postnatal care is defined as a care given to the mother and her newborn baby immediately after the birth of the placenta and for the first six weeks of life. Majority of maternal and neonatal deaths occur during childbirth and the postpartum period. Scaling up of maternal and newborn health through proper postnatal care services is the best way of reducing maternal and neonatal mortality.

**Method:**

A community based cross sectional study was conducted among 588 mothers who gave birth in the last one year from March 1–21; 2017. Systematic random sampling technique was used to select study participants. A pre-tested and structured questionnaire was used to collect the data. Data was entered in EPI info version 7 and analyzed using SPSS version 21. Logistic regression was applied to identify association between explanatory variables and the outcome variable. An adjusted odds ratio with 95% confidence interval and *p*-value less than 0.05 was computed to determine the level of significance.

**Result:**

A total of 588 participants were included in the analysis which was the response rate of 100%.

The prevalence of postnatal care service utilization in this study was 57.5%.

Maternal educational status of secondary school and above (AOR = 3.29, 95%CI: 1.94–5.57), family monthly income of above 1500 ETB (AOR = 2.85, 95%CI: 1.21–6.68), alive birth outcome of last pregnancy (AOR = 5.70, 95%CI: 1.53–21.216), planned and supported last pregnancy (AOR = 3.94, 95%CI: 1.72–9.01) and institutional delivery of last pregnancy (AOR = 3.08, 95%CI: 1.24–7.68) were positively associated with PNC service utilization.

**Conclusion:**

This study showed that the overall utilization of PNC service in Debretabour town is low. Mothers’ education, monthly income, last pregnancy birth outcome, wantedness of the pregnancy and place of delivery were significantly associated with postnatal care service utilization. To enhance PNC service utilization and reduce maternal and neonatal mortality women should obtain appropriate education. Furthermore all pregnant women should give birth in the health facilities.

## Background

The world Health Organization (WHO) stated that postnatal care (PNC) is defined as a care given to the mother and her newborn baby immediately after the birth of the placenta and for the first 42 days of life [1].

Majority of maternal and neonatal deaths occur during childbirth and postnatal period [2, 3].

The estimated maternal mortality ratio (MMR) declined across all MDG regions between 19,990 and 2015, although the magnitude of reduction varies substantially between regions [4].

According to 2015, the two regions with highest MMR are sub-Saharan Africa and Oceania. The MMR in developing regions was 19 times higher than in developed regions. Sub-Saharan Africa has the highest regional MMR (546) per 100,000 live births [4].

The 2016 Ethiopian Demographic and Health Survey (EDHS) showed that the MMR was 412 deaths per 100,000 live births. In other words for every 1000 live births about four women (4.12) died during pregnancy, childbirth or within six weeks of childbirth.

According to EDHS 2016, only 17% of women receive at least one postnatal care service from a health institution in the first 48 h after birth and Amhara region is the 3rd least region in postnatal care service utilization [5].

Utilization of maternal health care services decreases maternal and child mortality.

Postnatal care particularly prevents most maternal and child morbidity and mortality. Care given in the postpartum period assists health care providers to detect post- delivery problems and to give treatments timely [3, 6]. Shortage of care during this period could result in ill health, disabilities and deaths [3]. Around 65% of maternal deaths and 75% of newborn deaths occur in the first seven days after birth, and around half of these deaths occur in the first one day. A newborn is about 500 times more likely to die in the first day of life than at one month of age [7].

Appropriate postnatal care could save up to 60,000 newborn lives a year. In Ethiopia, if all new born receives appropriate postnatal care in the recommended time, neonatal mortality could be reduced by 10–27% [7].

Globally, there were an estimated 303,000 maternal deaths from complications related to pregnancy and childbirth in 2015 which is a decline of 43% from 1990. Majority of the maternal deaths occur in developing regions. It accounts for approximately 99% of the global maternal deaths in 2015, with Sub- Saharan Africa alone accounting for roughly 66% followed by southern Asia 22% [4].

Less attention was given for postnatal period in developing countries; women and their newborns don’t receive postnatal care services from a skilled birth attendant during the first days after childbirth [2].

Great majority of maternal and neonatal deaths occur during the first 2 days after childbirth. Hence, postnatal care for the women and the child is important in detecting and treating complications occur during the delivery as well as providing information for the mother on her and her newborn health [5].

To assess the extent of postnatal care utilization, EDHS 2016 asked respondents for their last birth in the 2 years preceding the survey, whether they had received a check up after delivery and the timing of the first checkup and it was found that the level of postnatal care coverage was low in Ethiopia. Only 17% of women reported having received a PNC checkup in the first 2 days after birth. In the case of the Amhara region percentage of women with a postnatal checkup in the first two days after birth was18.4% [5].

## Methods

### Study area and period

This study was conducted from March 1st to 21th, 2017, at Debretabour town. Debretabour, the city of South Gondar zone is found in the northern part of Amhara regional state 98 Kilometers from Bahirdar, the main city of Amhara regional state and 666 Kilometers away from Addis Ababa, the capital city of Ethiopia. According to the 2015 population projection estimate, there were 55,596 residents and around half of them were females. There are 3 health centers, 4 health posts, and 1 general hospital providing postnatal care services in the town.

### Study design and population

A quantitative research involving community based cross-sectional study was conducted among randomly selected reproductive age group of mothers who gave birth in the past one year preceding the data collection period.

Mothers who lived less than six months in the study area at the time of interview and who had communication problem were excluded from the study.

### Sample size calculation and sampling procedure

The single population proportion formula was used to calculate the sample size considering the following assumptions: proportion of women using postnatal care services 33.5% [8], 95% confidence level, 4% margin of error (absolute level of precision).

$$ {\displaystyle \begin{array}{c}\mathrm{Thus},\mathrm{n}=\left[\mathrm{Z}\ \alpha /{2}^{\ast }\ \mathrm{P}\ \left(1-\mathrm{P}\right)\right]/\mathrm{d}2\\ {}=1.{96}^{2\ast }\ 0.335\left(1-0.335\right)/0.0{4}^2\\ {}=535\end{array}} $$

Adding 10% non-response rate, a total sample size of 588 women were included.

Debretabour town intentionally was selected as a study site by considering the health status of women in the town and lack of previous researches related to maternal health care services. Systematic sampling technique was used to select the study units. From the four kebeles all kebeles were selected. The calculated sample size was distributed across the four kebeles proportionally to the size of the households in each kebele. Then the proportion of eligible mothers those who gave birth in the last one year prior to this study were identified. Based on this assumption the available data from registry of the local health extension workers they recently conducted the survey for immunization program was used. The sampling interval of households in each kebele was determined by dividing the total number of households to the allocated sample size. The initial household to be interviewed was selected randomly from the kebele house number registry using a number between 1 and the sample interval. The subsequent household to be included in the study was identified systematically through house-to-house visit. For households with more than one eligible woman, interview was done for one of the mothers using simple random sampling technique. Revisits of two to three times were made in case where eligible respondents were not available at the time of the survey by asking the neighbors whether an eligible women in that house was present or not, after all they were considered as non-respondents.

### Data collection tools and procedures

Data was collected by face to face interview using a structured and pre-tested questionnaire. The questionnaire first prepared in English and translated to Amharic, and then back to English. Four 3rd year diploma midwife students were used to collect data. Two BSC midwives were assigned to supervise the data collection process. Both the data collectors and supervisors were given one day training before the actual work about the aim of study, procedures and collection techniques going through the questionnaires question by question, art of interviewing and ways of collecting the data.

### Data quality control

The quality of data was assured by proper designing and pre-testing of the questionnaires in one of the Kebeles other than Debretabour, in Woreta town on 30 participants. Every day after data collection, questionnaires were reviewed and checked for completeness by the supervisor and principal investigator and the necessary feedback was offered to data collectors in the next morning and before ending all session incomplete questions were completed using precoded for controlling errors during data analysis.

### Data processing and analysis

The entire questionnaire were checked, coded and entered into EPI Info version 7 and exported to SPSS version 21 software. For analysis descriptive statistical procedures were utilized. Descriptive statistics like percentage, mean and standard deviation were used for the presentation of demographic data and magnitude of post natal care service utilization. Tables and graphs were also used for data presentation.

Binary logistic regression was used to identify factors associated with PNC service utilization on mothers. Variables with *P*-value less than or equal to 0.2 were selected in to multiple logistic regression models for controlling the possible effect of confounders and finally variables which had independent association with PNC service utilization were identified on the basis of AOR, with 95%CI and *p*-value less than 0.05.

### Ethical consideration

Ethical clearance was obtained from institutional Ethical Committee of Bahirdar University. Formal letter of cooperation was written for Regional Health Bureau, Debretabour health office and respective kebeles and permission was obtained. Written informed consent was obtained from each study subjects, each respondent was informed about the objective of the study that it contribute to improve maternal health. Any mother who was not willing to participate in the study has not been forced to participate. They were also informed that all data obtained from them would be kept confidential by using codes instead of any personal identifiers and is meant only for the purpose of the study. Health education on postnatal care service and related complication and other information was given for the participants during interview.

## Results

### Socio demographic characteristics of the participants

The total of 588 women participated in the study making the overall response rate 100%. More than half 330(56.1%) of the respondents were in the age group 20–29 with the mean ± SD 27.35 ± 5.48 years. Five hundred twenty one (88.6%) of them were married and 564(95.9%) of them were Orthodox Christian followers. Nearly one-six, 102(17.3%) of the respondents were unable to read and write and 260(44.2%) of them attend secondary school and above. Concerning their husbands’ educational status, 337(57.3%) of them were attended secondary school and above, and 269(45.7%) of respondents’ husband were government employed by occupation. The total monthly household’s income was ranging from 100 to 16,000 ETB while 74% of the participants had earned above 1500 ETB per month. Majority, 530 (90%) of the respondents had either TV and/or Radio in their houses (Table 1).

### Obstetric characteristics of respondents

Among the total respondents, 347 (59.0%) mothers were categorized as Para two to Para four. Of these, 18(3.1%) of mothers faced stillbirth while they gave the last birth.

Three hundred ninety- six (67.3%) of the current pregnancy were planned and supported. Almost all, 567(96.4%) of the mothers had antenatal care follow-up during the last pregnancy while 559(95.1%) of mothers gave their last birth at health institution.

Regarding to the mode of delivery, most respondents 432(77.28%) delivered by spontaneous vaginal delivery (Table 2).

**Table 1:** Socio-demographic characteristics of study participants at Debretabour town, March 2017(*n* = 588)

Variables	Frequency	Percent
Age		
< 20	61	10.4
20–29	330	56.1
30–39	173	29.4
40–49	24	4.1
Marital status		
Married	521	88.6
Single	32	5.4
Divorced	25	4.3
Widowed	10	1.7
Religion		
Orthodox	564	95.9
Muslim	18	3.1
Protestant	6	1.0
Ethnicity		
Amhara	580	98.6
Agew	8	1.4
Education		
Cannot read and write	102	17.3
Can read and write	68	11.6
Elementary education	158	26.9
Secondary and above	260	44.2
Occupation		
House wife	288	49
Government employed	139	23.6
Merchant	92	15.6
Daily laborer	42	7.1
Farming	5	0.9
Private employ	22	3.7
Husband education (*n* = 521)		
Cannot read and write	22	4.22
Can read and write	41	7.87
Elementary education	121	23.22
Secondary and above	337	64.68
Husband occupation (n = 521)		
Merchant	150	28.8
Farming	20	3.84
Government employed	269	51.63
Daily laborer	58	11.13
Driver	24	4.61
Average monthly income		
< 500 Eth birr	31	5.3
500–1500 Eth birr	121	20.6
> 1500 Eth birr	436	74.1

**Table 2:** Obstetric characteristics of the women who gave birth in the last 12 months in Debretabour town, Northwest Ethiopia, March 2017(n = 588)

Variables	Frequency	Percent
Parity		
1	203	34.5
2–4	347	59.0
5 and above	38	6.5
Outcome of birth		
Alive	570	96.9
Still birth	18	3.1
Nature of last pregnancy		
Planned and supported	396	67.3
Unplanned but supported	155	26.4
Unplanned and unsupported	37	6.3
Place of delivery		
Home	29	4.9
Health institution	559	95.1
Mode of delivery (*n* = 559)		
Spontaneous vaginal delivery	432	77.28
Instrumental delivery	83	14.85
Cesarean section	44	7.87
Advise for any danger signs before discharge (*n* = 559)		
Yes	375	67.08
No	184	32.92
ANC visit during last pregnancy(n = 588)		
Yes	567	96.4
No	21	3.6
Awareness about PNC service		
Yes	379	64.5
No	209	35.5

**Table 3:** Logistic regression analysis of factors associated with postnatal care utilization of the respondents in Debretabour, Ethiopia, 2017(n = 588)

Variables	Postnatal care utilization		COR(CI)	AOR(CI)	*p*-values
Marital status	Yes	No			
Married	310	211	3.23 (1.50–6.96)	1.27 (0.44–3.68)	
Divorced	13	12	2.38 (0.80–7.04)	2.61 (0.77–8.85)	
Widowed	5	5	2.20 (0.51–9.35)	1.31 (0.24–6.96)	
Single	10	22	1.00	1.00	
Educational status of women					
Unable to read and write	38	64	1.00	1.00	
Can read and write	35	33	1.78 (0.95–3.32)	1.70 (0.88–3.28)	
Primary education	80	78	1.72 (1.03–2.87)	1.47 (0.86–2.53)	
Secondary education and above	185	75	4.15 (2.56–6.73)	3.29 (1.94–5.57)	0.000
Monthly income					
< 500 ETB	9	22	1.00	1.00	
500–1500 ETB	55	66	2.03 (0.86–4.78)	2.18 (0.88–5.41)	
> 1500 ETB	274	162	4.13 (1.85–9.19)	2.85 (1.21–6.68)	0.016
Decision making power on her health					
Self	52	55	1.47 (0.71–3.07)	1.75 (0.64–4.78)	
Both	270	170	2.48 (1.28–4.78)	1.59 (0.69–3.68)	
Husband	16	25	1.00	1.00	
Decision making power on her child health					
Self	38	51	0.88 (0.45–1.73)	0.99 (0.47–2.10)	
Both	274	168	1.94 (1.11–3.38)	1.36 (0.73–2.52)	
Husband	26	31	1.00	1.00	
Parity					
One	122	81	2.07 (1.02–4.18)	1.41 (0.61–3.24)	
Tow-four	200	147	1.87 (0.94–3.68)	1.16 (0.53–2.51)	
Five and above	16	22	1.00	1.00	
Birth outcome of the last pregnancy					
Alive	335	235	7.12 (2.04–24.89)	5.70 (1.53–21.21)	0.009
Still birth	3	15	1.00	1.00	
Nature of the last pregnancy					
Planned and supported	236	160	4.58 (2.10–9.98)	3.94 (1.72–9.01)	0.001
Unplanned but supported	93	62	4.66 (2.06–10.56)	4.40 (1.84–10.52)	0.001
Unplanned and unsupported	9	28	1.00	1.00	
Place of delivery					
Home	7	22	1.00	1.00	
Health facility	331	228	4.56 (1.91–10.85)	3.08 (1.24–7.68)	0.015

### Proportion of postnatal care utilization

From total respondents, 338(57.5%) mothers were utilized postnatal care services with confidence interval of (53.4–61.6). Regarding to the frequency of postnatal care visit, 228(67.5%) of participants had visited once, 63(18.6%) women had two times, and the remaining 47(13.9%) were having three or more. The proportion of postnatal care visit within 24 h, at 3–7 days and at six weeks of postpartum were 103(30.5%),104(30.8%) and 162(47.9%) respectively.

Concerning types of service utilization during the postnatal visit, one-hundred sixty seven (28.4%) of the respondents utilized family planning, nearly half (44.9%) of them received immunization, 22.6% of them were tested and counseled for HIV and 106(18%) of them was counseled on breast feeding.

### Reasons for nonutilization of PNC services

**Fig. 1:**
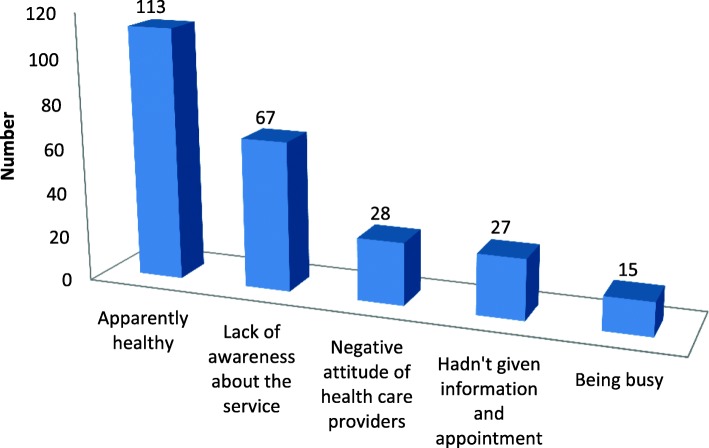
Reasons for not attending postnatal care services given by the study participants at Debretabour town, North West Ethiopia, March 2017(*n* = 250)

Different reasons were given by the participants for not attending postnatal care services. The most common reason mentioned by the participants for none utilization of postnatal care service was being apparently healthy (Fig. 1).

### Associated factors of postnatal care utilization

During bivariable logistic regression analysis, those variables that had significant association were marital status, respondents educational level, monthly income, decision making style of the women on her health, decision making style of the women on her child health, parity, birth outcome of the last baby, nature of the last pregnancy and place of delivery.

In multiple logistic regression analysis all variables with *p* values less than 0.2 were entered. Only educational status of the respondents, monthly income, birth outcome of the last pregnancy, nature of the last pregnancy and place of delivery were had significant association with postnatal care service utilization.

Accordingly, those women whose educational status secondary school and above were 3 times (AOR = 3.29, 95% CI: 1.94–5.57) more likely to utilize PNC service than those women who couldn’t read and write. Those mothers who had monthly household income greater than 1500 ETB were 2.8 times more likely to utilize PNC services than those women who earned less than 500 ETB (AOR = 2.85, 95%CI:1.21–6.68).Mothers who gave birth alive neonate were 5.7 times more likely to get postnatal care services than mothers who gave still birth(AOR = 5.70,95%CI:1.53–21.21).

The odds of having postnatal care visit for those women whose pregnancy were planned and supported were 3.9 times more likely to have PNC visit than those women whose pregnancy were unplanned and unsupported(AOR = 3.96,95%CI:1.72–9.01).

Those mothers who gave birth to their latest child at the health institution were 3 times (AOR = 3.08, 95%CI: 1.24–7.68) more likely to get postnatal care service utilization when compared with those mothers who gave birth to their latest child at home (Table 3).

## Discussion

This study indicated that more than half, 338(57.5%) of the participants had received PNC services with confidence interval of (53.4–61.6).

The utilization of postnatal care service in this study is lower than a study done in Bahi District, Tanzania 70.8% [9]. The possible explanation for this difference may be due to social context variation.

This result is also lower than the previous Ethiopian studies: Addis Ababa, 65.6% [10], Gondar Zuria District, 66.83% [11] and Adwa town 78.3% [12]. The possible explanation for this difference might be due to those communities with a low concentration of educated and poor women lead to the lower attention of the need for postnatal care service utilization. The other suggestion might be due to sample size determination differences. Lastly it might be due to lack of information about postnatal care, low maternal knowledge about danger signs of postnatal care and low attention of health professionals in counseling the women to came back for postnatal care.

According to this study the utilization of postnatal care service is higher than a study conducted in rural area of Western Rajasthan, India 35.86% [13], Nepal 43.2% [14], Palestine 36.6% [15], Soroti district Eastern Uganda 15.4% [16], Africa 36% [17] developing countries 36% [18], a study done in four Sub-Saharan African countries: Burkina Faso 25%, Kenya 33%, Malawi 41% and Mozambique 40% [19].The possible reason to the discrepancies might be due to cultural differences, time differences of study socioeconomic status, geographical factors, heterogeneity of study population and political concern of governments. The other possible explanation for the difference might be due to the unique nature of Ethiopia utilizing health extension workers.

This finding is also higher than the 2016 EDHS national and Amhara regional report [5].The possible reason for this difference may be the study area where EDHS included both women who lives in urban and rural areas of the country while our study includes only urban residents. Hence, women who live in urban areas are at a greater advantage of getting education opportunity and maternal health care services. Additionally, women in the urban areas may get easy access to health facility and health professionals as compared to their counterparts.

Postnatal care utilization of this study is higher than the research carried out in Sidama zone (Southern Ethiopia) 37.2% [20], Debre Markos town 33.5% [8], Abuna Gindeberet District, Oromiya 31.7% [21],Hadiya zone, South Ethiopia 22.7% [22], Dembecha District 34.8% [23], baseline and end line postnatal care surveys in Amhara and Oromia region [24], Jabitena district, Amhara regional state, 20.2% [25] and Lemo Woreda 51.4% [26].This difference may be due to time difference between these studies, socioeconomic status, and cultural factor. The Other possible explanation for these differences may be increased governmental focuses from year to year in order to improve maternal and neonatal healths.

According to this study, the odds of PNC service utilization among women with secondary school and above by education were 3 times than those who couldn’t read and write. This finding is consistent with the study done in Nepal [14], Cambodia [27], rural area of Western Rajasthan, India [13], developing countries [18], Africa [17], Nigeria [28], Bahi District, Tanzania [9]**,** rural India [29], Entoto Fana health center, Addis Ababa [30], rural Haramaya District, Eastern Ethiopia [31], southern Ethiopia [20], Abi-Adi Town, Tigray [32], Jabitena district, Amhara region [25] and Dembecha District, North West Ethiopia [23].The possible reasons for this similarity might be due to the fact that once a women is educated, her autonomy and decision making skill on her health and maternal health care services utilization is high [32].Similarly, uneducated mothers have no chance to participate in different social and economic positions, decision making and women’s empowerment. Additionally, education helps to increase mothers’ level of awareness and their acceptance of new idea and provides better education to other women regarding postnatal care services utilization.

In the present study, the odds of PNC service utilization among women with monthly income greater than 1500 ETB were 2.8 times than those who earn less than 500 ETB. This finding is consistent with a study done in developing countries [18], Rwanda [33], Nigeria [28], Tanzania [34], India [29], Nepal [14] and Addis Ababa, Ethiopia [30].

The possible suggestion for this might be women having better monthly income can afford for all expenses like for transportation.

The analysis also showed that, the odds of PNC service utilization among women with alive birth outcome were 5.7 times than those who gave still birth. This is in line with a study done in Debre Markos town [8]. The possible reason for this similarity may be good birth outcome might have better insight in postnatal care service utilization.

This study also showed that, the odds of PNC service utilization among women with desire of pregnancy were 4 times than those whose pregnancy was unplanned and unsupported. This finding is supported by a study done in three rural districts of Tanzania [35] and California [36].

This study also revealed that the odds of PNC service utilization among women who gave birth at health facility were 3 times than those who delivered at home. This finding is consistent with a study done in three rural districts of Tanzania [35], Rwanda [33], Nigeria [28], Tanzania [34], Nepal [14]**,** Addis Ababa, Ethiopia [30], Hadiya zone, South Ethiopia [22], Debre Markos town [8], Lemo Woreda, Ethiopia [26] and another study done in Jabitena district, Amhara region [25].The possible explanation for the similarity between place of delivery and postnatal care services utilization can be evidenced by women who gave birth in health institutions have better opportunity to receive health education related to postnatal care services, get access on benefits and availabilities of PNC services during their stay in health facilities.

### Limitation of the study

There could be recall bias since the women were asked for events within the last one year prior to the study. On the other hand the study did not include the rural population which is restricting the scope and relevance. Lastly, since the the design is quantitative it doesn’t address cultural issues of the respondents.

## Conclusion

This study demonstrated that utilization of postnatal care service is still low.

Educational status of the women, monthly household income, birth outcome of the latest pregnancy, wantedness of the last pregnancy and place of delivery were found to be statistically significant for the current PNC service Utilization.

## Abbreviations

EDHS: Ethiopian demographic health survey
MDG: Millennium development goal
MMM: Maternal mortality ratio
PNC: Postnatal care
WHO: World health organization
