# Correction to: Postnatal care service utilization and associated factors among women who gave birth in Debretabour town, North West Ethiopia: a community- based cross-sectional study

**DOI:** 10.1186/s12884-020-03019-2

**Published:** 2020-05-19

**Authors:** Kihinetu Gelaye Wudineh, Azezu Asres Nigusie, Shumiye Shiferaw Gesese, Azimeraw Arega Tesfu, Fentahun Yenealem Beyene

**Affiliations:** Department of Midwifery, College of Medicine and Health Sciences, Bahir Dar University, Bahir Dar, Ethiopia

**Correction to: BMC Pregnancy and Childbirth (2018) 18:508**

**https://doi.org/10.1186/s12884-018-2138-x**

Following publication of the original article [1], the authors identified an error in the author name of Azimeraw Arega Tesfu.

The incorrect author name is: Azimeraw Arega Tesu

The correct author name is: Azimeraw Arega Tesfu
